# Supplementary material for: Cloud BioLinux: pre-configured and on-demand bioinformatics computing for the genomics community
Source: BMC Bioinformatics. 2012 Mar 19;13:42. doi: 10.1186/1471-2105-13-42 (PMC3372431; doi:10.1186/1471-2105-13-42)
Supplement: Additional file 1 — Supplementary 1 Cloud BioLinux software documentation in the form of a mini, self-contained website. Users need to download and uncompress the .zip file, and open through a web browser the "index.html" file available on the main directory. (ZIP 1823 kb). [file 1471-2105-13-42-S1.ZIP › Cloud-BioLinux-Package-Documentation/docs/R Commander.html]

Bio-Linux Software Documentation Pages

Back to search form

## R Commander

|  |  |
| --- | --- |
| Name | R Commander |
| Description | **R Commander**  (aka Rcmdr) is a basic graphical interface for the R statistical software.   The basic Rcmdr system can be run by starting R on your Bio-Linux machine by typing `R` on the command line, and then loading the Rcmdr library: `library(Rcmdr)` Alternatively, you can run Rcmdr with an additional menu of Biodiversity analysis functions, as provided by the BiodiversityR and vegan packages. You can access documentation about Rcmdr by going to the help menu of the application when it is running. |
| Homepage | http://socserv.mcmaster.ca/jfox/Misc/Rcmdr/ |
| Remote Documentation | http://www.stats.bris.ac.uk/R/web/packages/Rcmdr/index.html      http://www.r-project.org/doc/Rnews/Rnews\_2007-3.pdf |
